# Involvement of professionals in research: knowledge integration, development of practice, and challenges: a group concept mapping study

**DOI:** 10.1186/s12961-021-00763-5

**Published:** 2021-08-11

**Authors:** Christine E. Laustsen, Pia Petersson, Albert Westergren, Maria Haak

**Affiliations:** 1 Research Platform for Collaboration for Health, Faculty of Health Sciences, Kristianstad University, Kristianstad, Sweden; 2 Department of Health Sciences, Faculty of Medicine, Lund University, Lund, Sweden

**Keywords:** Group concept mapping, Health personnel, Healthy aging, Stakeholder participation

## Abstract

**Background:**

Research and practice are often considered as two different worlds with different values, which causes a gap between them. Involving professionals such as practitioners, managers, decision-makers, and policy-makers in research on ageing and health might address the gap between research and practice, strengthen the healthcare system, and increase older people’s possibilities for healthy ageing. The aim of this study was to conceptualize professionals’ involvement in research on ageing and health from the perspective of the professionals themselves.

**Methods:**

A mixed method called group concept mapping was used. Professionals with experience being involved in research on ageing and health participated in qualitative data collection through brainstorming sessions (*n* = 29) and by sorting statements (*n* = 29). Afterwards, they participated in a quantitative data collection by rating statements according to how much each statement strengthened practice (*n* = 30) and strengthened research (*n* = 28). Multidimensional scaling analysis and hierarchical cluster analysis were used to conduct quantitative analysis. Latent qualitative analysis was also conducted.

**Results:**

Analysis resulted in eight clusters which illustrated conceptual areas of professionals’ involvement in research projects. The qualitative latent construct of the cluster map resulted in the themes: challenges for professionals; prerequisites and professionals’ learning can contribute to development of practice; and integrated knowledge benefits older people. There was a strong correlation between what strengthens practice and research (*r* = 0.92).

**Conclusions:**

This study illustrates conceptual areas of professionals’ own perspectives on what their involvement in research can lead to. Their involvement may lead to knowledge being integrated, and the professionals may learn through their involvement, which can contribute to the development of practice. However, there can also be challenges that need to be handled when professionals are involved in research. The study can be useful for improving the understanding of and actual involvement of professionals in research, and for optimizing the involvement of professionals.

## Background

Collaboration between professionals (practitioners, managers, decision-makers, and policy-makers) and researchers may play an important role in bridging the existing gap between research and practice [1], in order to strengthen the healthcare system [2] as well as increase older people’s possibilities for healthy ageing [3]. Even though involving professionals in research on ageing and health may lead to relevant and applicable knowledge, the collaboration is also complex and challenging to navigate [4].

It takes a long time for research to be applied in practice [5]. Moreover, much research is not published [6] and therefore never available for practice or other researchers. Even when published, some research might not be useful in practice if the result lacks relevance for practice, for patients, and is difficult to implement [7]. The lack of usefulness of research in practice might be because researchers and professionals prioritize differently and have different opinions about what are relevant research questions and outcome measures [6]. Research and practice are often considered as two different worlds with different values, which can cause barriers to the collaboration between researchers and professionals [8]. However, it is also argued that research and practice are aligned, that is, not two opposite phenomena [9]. Thus, research and practice complete one another, and both give insights in their specific areas, all useful for addressing today's challenges of an increasing ageing population.

The fact that people are living longer and the number of older people is increasing [10] entail several societal challenges [11], and challenges for healthcare systems [12]. A gap between research and practice is a contributory factor to the concern that older people do not always receive the best available care [13] or possibility for healthy ageing [3]. Several factors in older peoples’ physical and social contexts, besides the biological changes that happen when ageing, affects their potential for healthy ageing [14]. Hence, the consequences of the increasing older population and the gap between research and practice are complex challenges which cannot be solved by research or practice separately. Complex challenges call for collaboration between several organizations, often on several levels, and with people from different disciplines. Also, involving the older people themselves in research is called for to better meet their specific needs [14]. This implies that the context in which older people are embedded is crucial to consider, since older people and their surrounding context have a reciprocal relationship, in that they influence each other [15]. Professionals are in the older people’s surrounding context. For example, professionals such as practitioners may provide a service for the older people, or professionals such as managers, decision-makers, and policy-makers can play a role in regulating and developing healthcare [16]. Hence, it can be assumed that by involving professionals in research within areas such as older people’s health and social care, rehabilitation, the supportive environment, and prevention strategies, research might better meet the needs of the older population and enhance their possibilities for healthy ageing.

Within health systems, it has been shown that the involvement of professionals in research can improve the process of care as well as health outcomes [1]. Professionals hold knowledge that can contribute to research [17, 18]. Their involvement in research may lead to useful knowledge, since the professionals can both ensure that the research focuses on relevant areas as well as consider the context in which the research is intended to be utilized [18, 19]. Their practical knowledge, which is linked to specific situations, gives them a unique insight into the context, meaning that they understand patterns that cannot be understood by outsiders. Therefore, professionals can be defined as mediators of context-specific knowledge [20]. Researchers’ scientific knowledge is often more general and can be used to explain how something works or how it can be understood on a more abstract level. When professionals are involved, the research is conducted *with* them and not *on* them [21]. This implies that knowledge exchange and knowledge co-creation can occur between professionals and researchers, and professionals’ context-specific knowledge and researchers’ scientific knowledge can both contribute to each other.

Different kinds of knowledge, such as scientific and practical knowledge, were described as early as 300 BC by Aristotle [22]. He described scientific knowledge (episteme) as general and theoretical, not exclusive to one person or situation but more universal. Practical wisdom (phronesis) as knowledge was described as know-how that builds on an understanding of a situation and one’s experience [22]. However, there can be a lack of acknowledgement of different kinds of knowledge and different views on which knowledge is justifiable, which can contribute to the gap between practice and research [9]. Nevertheless, these different perspectives and kinds of knowledge are by themselves inadequate for addressing complex problems. This is why a combined and pluralistic approach is needed [9]. The involvement of professionals in research can facilitate the transfer of scientific knowledge from research to practice, and thereby help professionals to understand and increase knowledge of their practice [23]. Furthermore, when professionals are involved in research on ageing and health, they can contribute their tacit and particular knowledge about the context, to the benefit of older people [24].

A scoping review [25] looking at healthcare professionals’ involvement in research showed that experiences of challenges and benefits was often investigated, but there was limited focus on the goal of bridging the gap between research and practice. Another review [26] examined the involvement of a wider group of people and also found a focus on experiences of negative and positive outcomes, and a lack of evaluation of the effectiveness of research codesign in bridging the gap. This indicates that the involvement of professionals in research is a complex and challenging area to investigate. Researchers’ perspective on what the involvement of professionals in research can lead to has been investigated [4]. Thus, there is a need to add to this knowledge by also investigating the professional’s perspective on what their involvement in research can lead to. Involving professionals in conceptualizing their experience can be helpful for understanding a complex area from their perspective. Therefore, the aim of this study was to conceptualize professionals’ involvement in research on ageing and health from the perspective of professionals themselves.

## Method

This study was conducted within the UserAge programme [27]. A goal within the programme is to increase knowledge about involvement of frail older people, informal carers, and professionals in research on ageing and health. Being a part of this programme, this study focuses solely on the involvement of professionals in research on ageing and health.

In this study it was considered valuable to involve professionals in the research process, to capture the breadth and depth of their experience, and systematically structure the data in order to conceptualize what involvement of professionals in research can lead to. Therefore, group concept mapping (GCM) was used [28]. GCM is a mixed method that combines qualitative and quantitative methods, and makes it possible to combine a participatory worldview with a pragmatic worldview [28]. It is a democratic method of involving participants, ensuring their voices are heard and their worldviews expressed. In GCM studies, the quantitative research phase is built on the qualitative phase, and thereby aligns with the exploratory sequential approach [4, 29, 30]. The qualitative data collection is conducted through brainstorming sessions that helps the participants to specify indicators of the content of the area. The participants then sort the statements into groups based on how they perceive them to relate to each other. Thereafter, quantitative analyses are conducted based on this sorting, which results in a map of defined conceptual areas. Conceptualization can make abstract thoughts and worldviews clear and defined by defining a concept and its content. Indicators of the content are specified and boundaries of the concept area are established [31]. The participants are also asked to rate the statements according to predefined questions. Furthermore, the map of conceptual areas can be qualitatively analysed by its latent content, making the method slightly different from the exploratory sequential approach [32].

### Ethical considerations

The participants received written and oral information about the project and signed an informed consent form. The participants were informed that their participation was voluntary, that they could end their participation at any time, and that all data would be handled confidentially. Information was provided about the procedure, including that the brainstorming sessions were group-based. This meant that the participants were not anonymous in this phase. However, the results are presented on a group level with no possibility of identifying individual persons. The participants received contact information on the first author and could make contact with any questions related to their involvement. The study was approved according to Swedish regulations of the Ethical Review Board in Lund, Sweden (Dnr: 2018/34).

### Procedure and sample

The study was conducted according to the following steps: the first planning phase, brainstorming; the second planning phase, organizing, analyses, interpretation, and use [28].

### The first planning phase

The focus prompt was developed during the first planning phase. Purposeful sampling was used [33]. Contact information of potential participants was obtained through researchers who had experience of involving professionals in research on ageing and health. The inclusion criteria were: professionals who, in relation to their work, had been involved in one or more parts of the research process; and the research in which they had been involved was in the field of ageing and health, e.g. older people’s health and social care, rehabilitation, and the supportive environment. The involvement could be in varying degrees, from consultation to collaboration, but was specified as being more than just being interviewed or answering a survey.

**Fig. 1:**
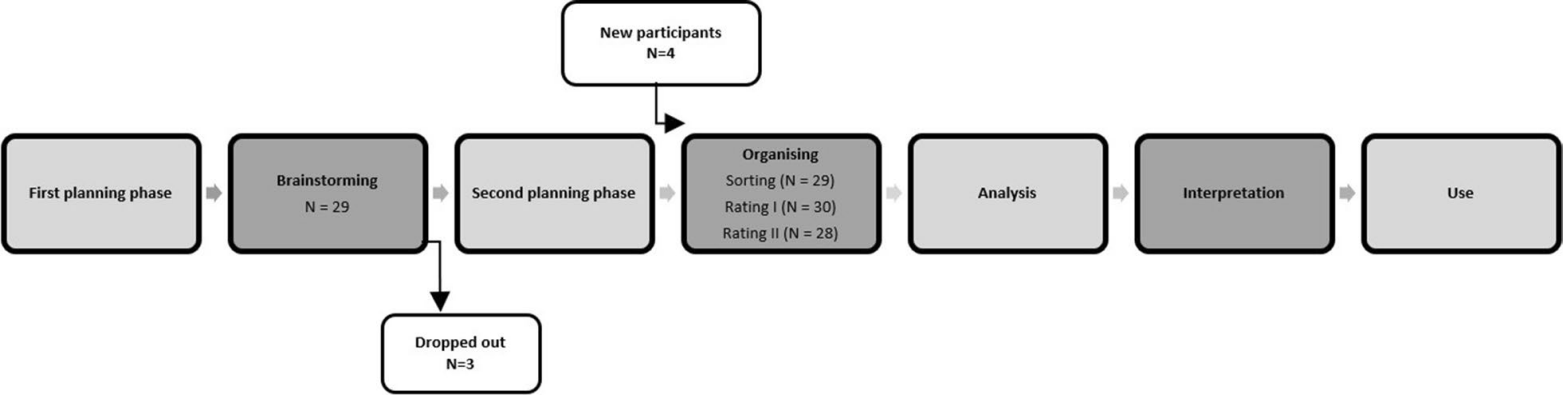
Overview of the GCM process and number of participants (*n*) in each step. The darker shading shows the steps involving the participants

An email containing information about the study was sent to 74 professionals asking if they were interested in participating. Twenty-nine persons declined to participate, mainly due to lack of time, and 12 persons did not respond. In total, 33 (45%) professionals agreed to participate in the study. See Fig. 1 for an overview of the GCM process and the number of participants in each step. There was a dropout of three participants after the brainstorming sessions, and four people who were interested in participating but were unable to take part in any brainstorming sessions participated in only the organizing step. GCM is a flexible method that allows for different people to participate in the different steps [34]. One participant did not complete the sorting of all the statements, and thus this sorting was not included. Furthermore, two participants dropped out during the organizing step.

Professionals who agreed to participate were asked to complete a questionnaire about age, gender, and work experience (Table 1). The professionals had experience of being involved in the following ways: member of a reference group or steering group; giving advice about the research; recruiting participants; collection or interpretation of data; disseminating results; or being a project leader. Most participants were women, in the age group of 41–50 years, worked in municipalities, and were mostly nurses, occupational therapists, or dieticians (Table 1).

**Table 1:** Characteristics of participants in the different steps of the GCM process

	Brainstorming	Organizing			Total
	*n* = 29 = (%)	Sorting *n* = 29 (%)	Rating I *n* = 30 (%)	Rating II *n* = 28 (%)	*n* = 33 (%)
Sex					
Women	23 (79.3)	22 (75.9)	23 (76.7)	22 (78.6)	25 (75.8)
Age, years					
18–30	2 (6.9)	2 (6.9)	2 (6.7)	2 (7.1)	2 (6.0)
31–40	4 (13.8)	5 (17.2)	5 (16.7)	5 (17.9)	5 (15.2)
41–50	10 (34.5)	10 (34.5)	11 (36.7)	10 (35.7)	12 (36.4)
51–60	7 (24.1)	7 (24.1)	7 (23.3)	7 (25.0)	7 (21.2)
61–70	6 (20.7)	5 (17.2)	5 (16.7)	4 (14.3)	7 (21.2)
Place of work					
Municipality	19 (65.5)	19 (65.5)	20 (66.7)	18 (64.3)	21 (63.6)
Region	9 (31.0)	9 (31.0)	9 (30.0)	9 (32.1)	11 (33.3)
Self-employed	1 (3.5)	1 (3.5)	1 (3.3)	1 (3.6)	1 (3.0)
Years of experience in current profession					
1–4	1 (3.5)	1 (3.5)	1 (3.3)	1 (3.6)	1 (3.0)
5–10	5 (17.2)	5 (17.2)	6 (20)	5 (17.9)	6 (18.2)
11–20	12 (41.4)	11 (37.9)	11 (36.7)	11 (39.3)	14 (42.4)
> 21	11 (37.9)	12 (41.4)	12 (40.0)	11 (39.3)	12 (36.4)
Profession^a^					
Dietician	6 (20.7)	6 (20.7)	6 (20.0)	6 (21.4)	6 (18.2)
Nurse	8 (27.6)	7 (24.1)	7 (23.3)	6 (21.4)	8 (24.2)
Occupational therapist	6 (20.7)	8 (27.6)	8 (26.7)	8 (28.6)	8 (24.2)
Physiotherapist	2 (6.9)	3 (10.3)	3 (10.0)	3 (10.7)	3 (9.1)
Other^b^	7 (24.1)	5 (17.2)	6 (20.0)	5 (17.9)	8 (24.2)

Rating I: Rates on a scale from 1 (not at all) to 4 (very much) the extent to which the statements can strengthen *practice* in research involving professionals

Rating II: Rates on a scale from 1 (not at all) to 4 (very much) the extent to which the statements can strengthen *research* involving professionals

^a^Six of the 33 participants worked as managers

^b^Assistant nurse, behavioural scientist, doctor, public health strategist, social scientist, and software developer

### Brainstorming

Twenty-nine professionals participated in brainstorming sessions. Eight brainstorming sessions were held between September 2019 and March 2020. Three sessions were conducted in person, and five were conducted via videoconferencing. Two to eight participants participated in each session. The participants brainstormed on the focus prompt: “*Involvement of professionals in research on ageing and health can lead to…*”, and were asked to freely brainstorm according to their experience of their involvement in research, as well as reflect on both positive and negative aspects of what their involvement led to in terms of practice as well as research. Following the rules for brainstorming, the participants were asked not to debate the statements [28]. In order to facilitate the brainstorming, two of the authors guided each session. Statements were written down and displayed directly on a screen for the participants to see.

### Second planning phase

All statements generated in the brainstorming session were reviewed and synthesized by the researchers during the second planning phase. The aim of this was to reach a manageable number of statements for the participants to sort and rank, and to ensure the representativeness of the participants’ experiences, and saturation of the topic [35]. The first author systematized the reviewing by arranging statements with the same or similar meaning or keywords on a horizontal row in a document. This allowed transparency and an audit trail of the process [36]. It enabled all the authors to be engaged in the process of finding the statement that best captured the meaning of all the statements in each row of the document. Statements not related to the focus prompt were marked and removed from the list by consensus among all the authors.

### Organizing

In the next step, the participants were asked to individually organize the statements that remained after the second planning phase, by sorting (*n* = 29) and rating (rating I, *n* = 30; rating II, *n* = 28) the statement using the web-based Concept System® groupwisdom™ (Concept Systems Inc, Ithaca, NY, USA). By using this system, the participants could conduct the sorting and rating when they wanted, within a time frame of 5 weeks.

The participants were asked to sort the statements into groups based on to how they perceived them to relate to each other, and then to label each group. After sorting the statements, the participants were asked to rate the statements according to the following two rating questions: “To what extent can the following statements strengthen *practice* when conducting research with the involvement of professionals?” (rating I) and “To what extent can the following statements strengthen the *research* conducted with the involvement of professionals?” (rating II). *Strengthening* was described for the participants as meaning developing relevant, applicable, and/or sustainable knowledge. The participants were asked to use a scale from 1–4 (1 = not at all, 2 = a little, 3 = a lot, 4 = very much).

### Analyses

The participants’ sorting was analysed using a multidimensional scaling (MDS) analysis and hierarchical cluster analysis. The Concept System® groupwisdom™ was used for the analysis. The system first calculates a similarity matrix, then MDS is used to place points on a two-dimensional map, illustrating how the statements are related based on the participants’ sorting of the statements into groups. The more frequently the statements have been sorted together, the closer they appear to each other on the point map [37]. The fit between the similarity matrix and the resulting point map is indicated by a stress value. In GCM studies, the stress value is usually somewhere between 0.10 and 0.35 [28], with an average stress value of 0.28 [35]; however, the lower the stress value, the better the fit. A sample between 20 and 30 persons in the sorting step is estimated to probably give the best fit between the participants’ sorting and the point map [35].

How the participants sorted the statements and how they believed the statements related to each other are of interest. The aggregated sorting by all the participants was calculated and is shown as the bridging value (BV) ranging from 0 to 1. A statement with a low BV has mostly been sorted with statements nearby. A statement with a high BV has been sorted with statements placed further away on the map. Low BV indicates that the statement is anchored to its place on the map. An *anchor statement* indicates the conceptual meaning of the cluster in which it is located. Higher BV indicates that the statement relates to other areas of the map, by bridging from its location to other clusters on the map. The BV for a cluster represents the average of all the statements’ BVs in each cluster, indicating whether it is a homogeneous cluster (low BV) or a more heterogeneous cluster (high BV) [28]. The BV of a cluster is illustrated graphically as layers of the cluster: the higher the BV of a cluster, the more layers there are on the map.

To group the statements into clusters, a hierarchical cluster analysis was used. The goal was to find an optimal number of clusters based on conceptual reasoning. Each statement illustrated as a point on the point map could have a potential to be its own cluster. The two points statistically closest to each other were then merged to form a cluster. In each stage, the two clusters in the closest proximity to each other were merged. The decision on the number of clusters relies on a qualitative analysis of the content of the clusters and the statements, and the BVs are also considered [28]. The optimal number of clusters and their labels were discussed and decided on by all the authors. To facilitate this decision, an inductive qualitative interpretation [38] of the clusters content was carried out by looking at the participants’ suggestions for labels, the statements in each cluster, and the statements with the lowest BV within each cluster.

The analysis of the participants’ mean rating of the two ratings (rating I and rating II) of the statements is illustrated in a go-zone map, which is a bivariate scatterplot. The go-zone makes it easier to compare the mean rating on each statement. The aggregated mean rating of all the statements in a cluster is illustrated in a pattern match. The pattern match makes it easier to compare the aggregated ratings at cluster level by a visualization in a ladder graph.

Qualitative analysis [38] of the latent construct of the cluster map was facilitated by inductively studying the cluster map, in combination with the go-zone map and the pattern match. A latent analysis can be used to see higher level areas and facilitate interpretation of the maps [39–41].

### Interpretation and use

The step of *interpretation* and *use* of the results can be conducted in collaboration with the participants or other relevant users of the research. To validate the interpretation of the results, member checks were conducted [36] at meetings with some of the participants, where the preliminary results were presented and discussed. The professionals said that they recognized their experiences in the results, and no changes to the results were suggested. How the results from this study can be used will be elaborated upon in the discussion and conclusion.

## Results

The brainstorming sessions generated 432 statements, which were reviewed and synthesized, resulting in a final list of 80 statements (Table 2).

**Table 2:** Eighty statements on what professionals’ involvement in research on ageing and health can lead to, within eight clusters

Cluster solution and statements	Bridging value^a^	Rating I practice^b^	Rating II research^c^
Cluster 1: Challenges for the professional in relation to practice	*0.16*	*2.12*	*1.99*
3 The presence of professionals inhibits older people from speaking	0.25	1.60	1.64
17 Challenges for professionals (involved in the research) in relation to their colleagues	0.10	2.40	2.04
22 The professionals become coordinators	0.33	2.50	2.26
25 Professionals feel divided between practice and research	0.05	2.47	2.14
27 Prestige for the professionals	0.27	1.97	1.89
28 Professionals may feel that they are in a vulnerable situation	0.00*	1.83	1.74
32 Extra tasks are assigned to professionals, which takes time from the practice	0.16	2.52*	2.14
41 Uneven distribution in which professionals are involved	0.02	2	1.78
42 Ethical challenges in the relationship between professionals and older people	0.44	2.03	1.96
43 Frustration for professionals in the case of absence of continued funding for research projects	0.10	2.10	2
44 Frustration for professionals while waiting for research results	0.06	1.90	1.75
61 Professionals influence research based on their own interests	0.07	1.77	1.82
69 Research projects take longer	0.17	2.10	2.07
79 Professionals’ considerations in the recruitment of participants affect the research project	0.19	2.45	2.67*
Cluster 2: Challenges for professionals in relation to research	*0.23*	*2.31*	*2.25*
26 Professionals act as interpreters between researchers and practice	0.27	2.43	2.54*
31 Professionals question the research	0.18*	2.30	2.39
33 Professionals’ preconceptions affect the results of the research	0.19	2.60	2.41
49 Researchers become "hostage" to practice	0.23	1.66	1.56
62 Challenges in the meeting between professionals and researchers due to lack of knowledge of each other’s starting points	0.22	2.27	2.32
65 Professionals need knowledge about scientific concepts	0.18*	2.63*	2.43
68 Ethical challenges arise in the relationship between professionals and researchers	0.35	2.27	2.11
Cluster 3: Prerequisites affecting professionals' involvement in research	*0.75*	*3.01*	*2.84*
19 Professionals gain confidence in their own ability and knowledge	0.48*	3	2.59
47 Management and colleagues gain an understanding of the professionals	0.97	2.70	2.54
48 Learning for the researcher	1.00	2.97	3.18*
51 Management should ensure conditions exist that facilitates the involvement of professionals	0.67	3.34*	3.11
74 The practices’ prerequisites influence the implementation of research results	0.66	3.03	2.79
Cluster 4: Professionals’ involvement increases their interest and engagement in R&D	*0.30*	*2.91*	*2.85*
24 Professionals become curious about getting training in research	0.25	2.77	2.82
34 Professionals’ efforts in research are made visible at the political level	0.54	2.62	2.68
35 Professionals develop a critical approach	0.19*	2.77	2.63
36 Increased interest in research among professionals	0.23	2.90	3.26*
38 Professionals inspire further development of practice	0.24	3.17	2.89
60 Adaptation of research to practice, through the knowledge of the professionals	0.46	2.97	3.11
76 Professionals are given the responsibility for implementation	0.19*	3.20*	2.57
78 Improved recruitment of participants	0.33	2.86	2.85
Cluster 5: Professionals contribute with their knowledge	*0.15*	*2.96*	*2.83*
16 A common foundation is created that strengthens the professionals as a group	0.15	3.10	2.61
18 Professionals acquire an understanding of research	0.25	3.11	3.07
21 Professionals feel involved in research	0.14	3.24	3.22*
23 Professionals can contribute with their knowledge	0.07*	3.30*	3.19
29 Professionals find practical solutions	0.09	3.20	3.04
30 Professionals have an influence on the research process	0.17	2.87	3.04
37 Professionals make researchers aware of ethical aspects	0.16	2.55	3.04
39 Professionals contribute with a critical approach	0.17	2.93	2.75
40 Professionals become spokespersons for the research	0.09	2.62	2.79
67 Ethical challenges can more easily be handled	0.22	2.66	2.64
Cluster 6: Bridging the gap between practice and research	*0.15*	*3.13*	*3.21*
9 Increased consensus between professionals and researchers	0.30	3.27	3.3
10 Mutual learning between professionals and researchers	0.25	3.33	3.36*
11 Professionals and researchers acquire an understanding of each other	0.26	3.23	3.29
12 Communication between professionals and researchers gains attention	0.25	2.90	3.15
13 Integration of research and practice perspectives	0.25	3.10	3.07
14 Conditions for research through networks are created	0.21*	2.93	3.04
15 Trust and confidence are required between all involved in the research project	0.35	3.07	3.3
46 Bridges are built between different organizations	0.29	3.10	3.07
55 A bridge is built between practice and research	0.29	3.23	3.32
59 Additional value for researchers and professionals	0.24	3.17	3.29
64 Dialogue between professionals and researchers is needed for consensus	0.31	3.37*	3.25
72 Research is de-dramatized	0.30	2.90	3.14
Cluster 7: Applicable research that benefits practice	*0.15*	*3.08*	*3.17*
20 Professionals feel commitment for research	0.10	3.17	3.15
50 Increased legitimacy for research projects	0.09	2.90	3.19
52 Researchers translate professionals’ expert knowledge into management and politics	0.22	2.90	3.04
57 A clearer picture of the problem is created through external monitoring	0.25	2.93	3.14
70 The research project becomes anchored in practice	0.19	3.40*	3.41*
71 More useful research results	0.13	3.07	3.26
73 More research questions are generated	0.10	2.83	2.93
75 Faster implementation of research results in practice	0.19	3.20	3.11
77 Dissemination of research in practice is facilitated	0.21	3.21	3.25
80 Better quality of collected data	0.04*	3.23	3.21
Cluster 8: Research that benefits older people	*0.16*	*3.13*	*3.08*
1 Prevention of ill health and improving the health of the older people	0.09	3.17	3.11
2 Situational information that contributes to learning among older people	0.14	2.93	2.86
4 Creation of relevant interventions for older people	0.06	3.30	3.14
5 The research is focused on the everyday needs of older people	0.17	3.47*	3.11
6 An increased understanding of the needs of the older people	0.09	3.33	3.29
7 Older people feel secure through trust in the professionals	0.28	3.14	3.07
8 Professionals enable older peoples’ voices to be heard	0.36	3.10	3.04
45 Evidence-based practice	0.11	3.17	3.26
53 The research contributes to a systematic approach in practice	0.05	3.03	2.96
54 Research has effect in practice independently of research results	0.04*	2.66	2.56
56 Research results are reported in popular science	0.53	2.87	3.04
58 Increased social benefits	0.14	3.40	3.43*
63 Research forms the basis for improvement work	0.13	3.24	3.30
66 Continuous development of practice	0.06	3.07	2.93

^a^Bridging value: the mean value for all the bridging values of statements within the cluster is shown in italics. Asterisks (*) show statements with the *lowest bridging value* within a cluster

^b^Rating I: Practice: mean rating on a scale from 1 (not at all) to 4 (very much), from the rating of the extent to which the statements can strengthen practice. The mean rating for all the statements within the cluster is shown in italics. Asterisks (*) show statements with the *highest rating value* within the cluster.

^c^Rating II: Research: mean rating on a scale from 1 (not at all) to 4 (very much) from the rating of the extent to which the statements can strengthen research. The mean rating for all the statements within the cluster is shown in italics. Asterisks (*) show statements with the *highest rating value* within the cluster

**Fig. 2:**
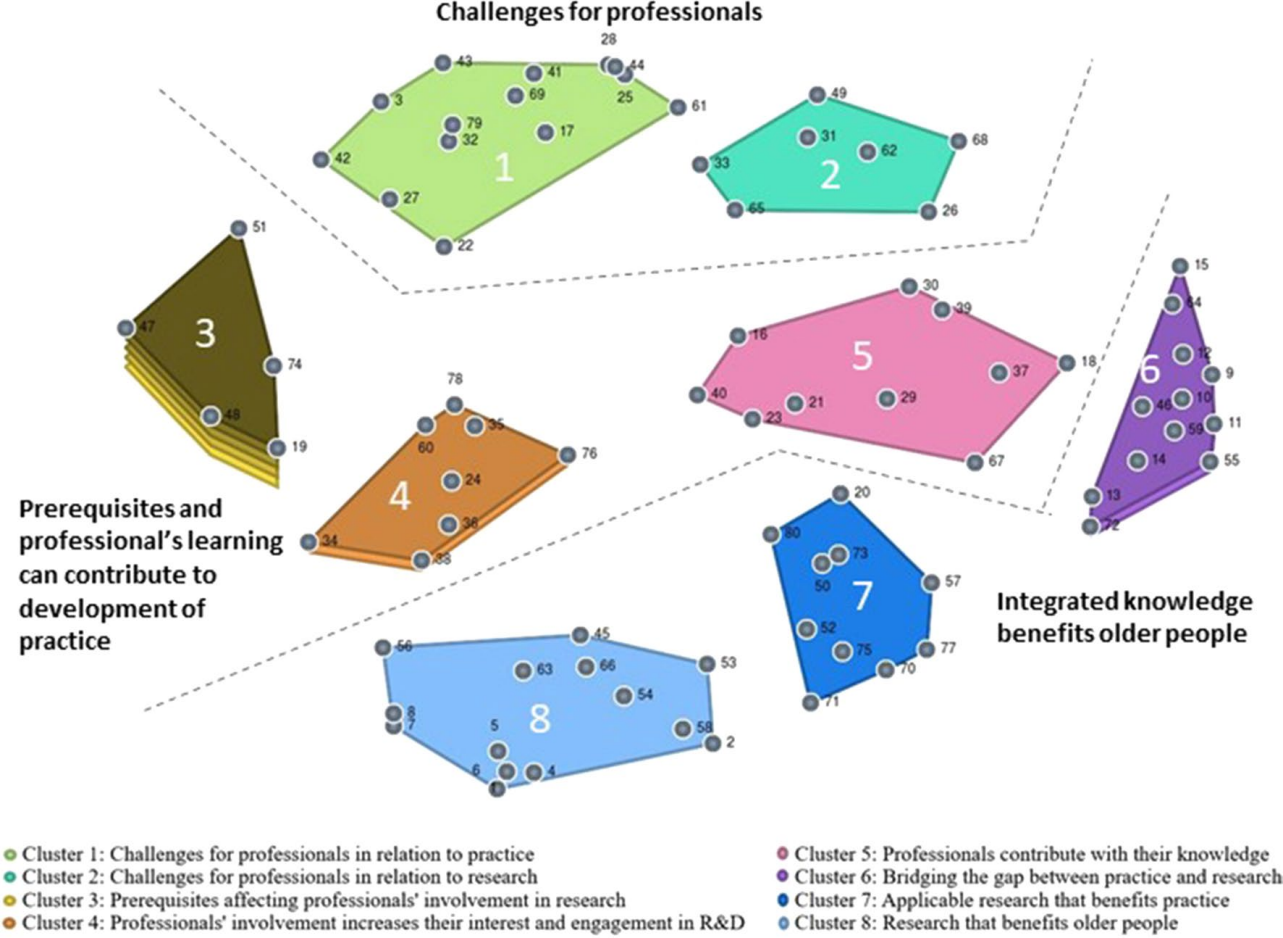
Point cluster map illustrating statements, conceptual areas, and themes

The MDS analysis of the participants’ sorting of statements is visually illustrated in the point cluster map (Fig. 2). Each point represents a statement, and the location of each point on the map represents the statement’s relation to the others. A stress value of 0.28 indicates an acceptable degree of fit between the similarity matrix and the point map. By conducting hierarchical cluster analysis, a solution of eight clusters was chosen (see Fig. 2).

The qualitative content of the clusters in this solution appeared most meaningful in relation to the focus prompt and the aim of the study. The cluster map’s eight clusters are as follows: challenges for professionals in relation to practice (cluster 1); challenges for professionals in relation to research (cluster 2); prerequisites affecting professionals’ involvement in research (cluster 3); professionals’ involvement increases their interest and engagement in research and development (R&D) (cluster 4); professionals contribute with their knowledge (cluster 5); bridging the gap between practice and research (cluster 6); applicable research that benefits practice (cluster 7); and research that benefits older people (cluster 8).

The BVs of the clusters and the statements are listed in Table 2 and illustrated in Fig. 2, showing that the cluster *prerequisites affecting professionals’ involvement in research* has a high BV (0.75), indicating that it is rather heterogeneous.

**Fig. 3:**
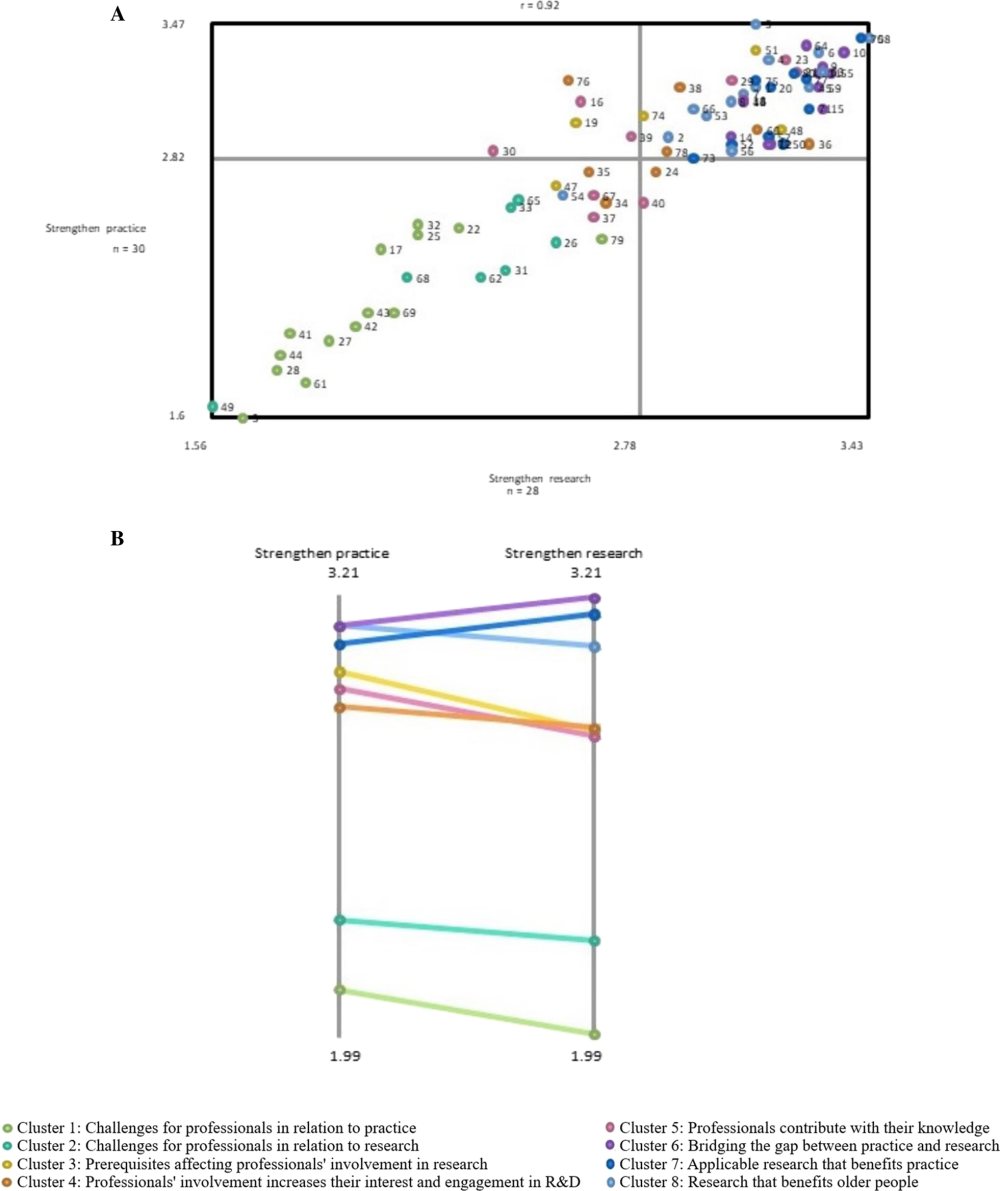
Illustrates the rating in terms of strengthening practice and research. **A** The go-zone map illustrates the mean of the participants’ ratings for each statement. *X*-axis (strengthens research), *Y*-axis (strengthens practice). **B** The absolute pattern match illustrates the average rating at a cluster level. The colours illustrate which cluster the statements belong to

The mean rating of the statements and aggregated mean rating of all the statements in a cluster are described in Table 2. The go-zone map (Fig. 3A) illustrates the mean rating of the statements plotted on the X-axis (strengthens research) and the Y-axis (strengthens practice). The go-zone map illustrates that the more a statement is graded to strengthen research, the more it is also graded to strengthen practice, meaning that there is a strong positive correlation (*r* = 0.92). The mean (range) of the rating of strengthening research is 2.78 (1.56–3.43). The mean (range) of the rating of strengthening practice is 2.82 (1.56–3.47). The statements in the go-zone, the upper-right quadrant, are rated high in terms of strengthening both research and practice. All the statements from cluster 6 (*bridging the gap between practice and research*), cluster 7 (*applicable research that benefits practice*), and cluster 8 (*research that benefits older people*), except statement 54, “Research has effect in practice independently of research results”, are in this zone. The lower-left quadrant, the “o-zone”, contains all the statements from clusters 1 (*challenges for professionals in relation to practice*) and 2 (*challenges for professionals in relation to research*). These statements were rated lower in terms of strengthening both research and practice. Statements from clusters 3, 4, and 5 are located more centrally in the go-zone map.

The absolute pattern match (Fig. 3B) illustrates the average rating at a cluster level in terms of strengthening practice and research. It also visualizes the relationship between the rating of the clusters and enables further assessment of the findings. In this study it contributed to the latent qualitative analysis.

By looking at the results, the cluster map, the go-zone map, and the pattern match, an inductive qualitative analysis resulted in three themes comprising the conceptual content of the clusters embedded. For an overview of themes and areas see Table 3.

**Table 3:** Overview of themes and areas

Theme	Conceptual areas
Challenges for professionals	Challenges for professionals in relation to practice Challenges for professionals in relation to research
Prerequisites and professionals’ learning can contribute to development of practice	Prerequisites affecting professionals’ involvement in research Professionals’ involvement increases their interest and engagement in R&D Professionals contribute with their knowledge
Integrated knowledge benefits older people	Bridging the gap between practice and research Applicable research that benefits practice Research that benefits older people

### Challenges for professionals

The theme *challenges for professionals* illustrates challenges professionals meet when they are involved in research on ageing and health. By being involved, they often stand between practice and research, which may lead to them feeling split in their role and being in a vulnerable position, as described in the two clusters *challenges for professionals in relation to practice* and *challenges for professionals in relation to research*.

#### Cluster 1

*Challenges for professionals in relation to practice* illustrates the challenging and vulnerable situation the professionals can be in when involved in research. One of the challenges for the professionals is the feeling of standing between the researcher group and the workplace colleagues. The different worldviews and values of these groups can make the professionals feel divided in their allegiance. Challenges can also relate to ethical aspects for professionals when acting as intermediaries between the researchers and the older people. Other challenges are that the professionals’ own interests might influence the research, or the research project might be affected by the professionals' considerations concerning the recruitment of participants. Though it can be prestigious for the professionals to be involved, they may also feel frustration while waiting for research results and when continued funding for research projects is not available.

The cluster has an average BV of 0.16. Statement 28, “Practitioners may feel that they are in a vulnerable situation”, has the lowest BV (0.00), being the anchor of the cluster. The statements in the cluster are rated low in terms of strengthening research and practice. Statement 32, “Extra tasks are assigned to professionals, which takes time from the practice”, was rated highest in terms of strengthening practice (2.52), and statement 79, “Professionals' considerations in the recruitment of participants affect the research project”, was rated highest in terms of strengthening research (2.67).

#### Cluster 2

*Challenges for professionals in relation to research* illustrates challenges the professionals can meet regarding the relationship between themselves and the researchers. Some ethical challenges arise in this relationship due to the two groups’ different kinds of knowledge and different understandings of each other’s real-world situations. When professionals act as interpreters between researchers and practice, it can lead to challenges, for instance if the professionals' preconceptions affect the results of the research, if they lack knowledge about scientific concepts, or if they question the research. The challenging relationship between professionals and researchers may even lead to researchers becoming "hostages" to practice.

The cluster has an average BV of 0.23. Two statements in this cluster have the lowest BV (0.18): statement 31, “Professionals question the research”; and statement 65, “Professionals need knowledge about scientific concepts”. Statement 65 was also rated highest in terms of strengthening practice (2.63), and statement 26, “Professionals act as interpreters between researchers and practice”, was rated highest in terms of strengthening research (2.54).

### Prerequisites and professionals’ learning can contribute to development of practice

This theme illustrates prerequisites for being involved in research on ageing and health, and the professionals’ opportunity to learn. The theme includes the following clusters: *prerequisites affecting professionals’ involvement in research*; *professionals’ involvement increases their interest and engagement in R&D*; and *professionals contribute with their knowledge.*

#### Cluster 3

*Prerequisites affecting professionals’ involvement in research* shows that the management and the practices prerequisites influence learning possibilities and the development of practice. By being involved, professionals gain confidence in their own ability and knowledge. Prerequisites affecting the involvement were for instance management and colleagues showing understanding, and management ensuring conditions exist that facilitate the involvement. A prerequisite for the professionals’ involvement was the researcher’s willingness to learn in the process of collaboration. Prerequisites that affect professionals’ involvement can also be that the professionals believe in themselves as capable of being involved in research and are able to contribute with their knowledge.

The cluster has an average BV of 0.75, which illustrates that prerequisites are strongly related to the other clusters, and it is also the most heterogeneous cluster. Statement 19, “Professionals gain confidence in their own ability and knowledge”, has the lowest BV (0.48). Statement 51, “Management should ensure conditions exist that facilitate the involvement of professionals”, was rated highest in terms of strengthening practice (3.34), and statement 48, “Learning for the researcher”, was rated highest in terms of strengthening research (3.18).

#### Cluster 4

*Professionals’ involvement increases their interest and engagement in R&D* illustrates the development that happens when professionals are involved in research. When involved, professionals become interested in learning more about research, develop a critical approach, and may inspire further development of practice. Their interest in R&D projects increases when they find that they can contribute with their knowledge and can facilitate the adaptation of research to practice.

The cluster has an average BV of 0.30. The two statements with the lowest BV (0.19) are statement 35, “Professionals develop a critical approach” and statement 76, “Professionals are given the responsibility for implementation”. Statement 76 was also rated highest in terms of strengthening practice (3.20), and statement 36, “Increased interest in research among professionals”, was rated highest in terms of strengthening research (3.26).

#### Cluster 5

*Professionals contribute with their knowledge* shows some positive aspects of the involvement of professionals. The professionals acquire an understanding of research and feel involved. Therefore, they can contribute with their knowledge and their critical approach, and can influence the research process. For example, they can find practical solutions to issues in the project, make the researchers aware of ethical aspects, and some ethical challenges can be more easily handled by involving professionals.

The cluster has an average BV of 0.15, and statement 23, “Professionals can contribute with their knowledge”, has the lowest BV (0.07). This statement was also rated highest in terms of strengthening practice (3.30). Statement 21, “Professionals feel involved in research”, was rated highest in terms of strengthening research (3.22).

### Integrated knowledge benefits older people

This theme shows the benefits that involvement of professionals in research on ageing and health might lead to. There are gains for the professionals, the researchers, the practice, as well as the research, which leads to benefits for the older people enhancing their potential for healthy ageing.

#### Cluster 6

*Bridging the gap between practice and research* illustrates how the perspectives from research and practice become integrated. Mutual learning and understanding between professionals and researchers occurs when professionals are involved. When professionals learn and understand research, it becomes de-dramatized. However, to reach consensus requires trust and confidence between the professionals and the researchers, and a dialogue. The network and collaboration between professionals and researchers create conditions for research which might otherwise be difficult to conduct. There are also bridges built between different organizations which are involved in the research. Taken together, this all adds value to both the researchers and the professionals.

The cluster has an average BV of 0.15. Statement 14, “Conditions for research through networks are created”, has the lowest BV (0.21). Statement 64, “Dialogue between professionals and researchers is needed for consensus”, was rated highest in terms of strengthening practice (3.37), and statement 10, “Mutual learning between professionals and researchers”, was rated highest in terms of strengthening research (3.36).

#### Cluster 7

*Applicable research that benefits practice* shows how the involvement of professionals may make the research more beneficial for practice and increase the legitimacy of the research project. When involving professionals, their knowledge and monitoring of the practice creates a clearer picture of the problem that is the focus of the research, and this might generate more research questions. Also, when they are involved in collecting data, it may be of better quality, giving more useful research results. The research projects become more anchored in practice, leading to faster implementation of research results and facilitation of the dissemination of the research. The professionals’ commitment to research must be supported by the researchers, who can translate the professionals’ expert knowledge into management and politics.

The cluster has an average BV of 0.15. Statement 80, “Better quality of collected data”, has the lowest BV (0.04). Statement 70, “The research project becomes anchored in practice”, was rated highest in terms of strengthening practice (3.40) and in terms of strengthening research (3.41).

#### Cluster 8

*Research that benefits older people* illustrates different benefits for the older people that the research may lead to when it is based on knowledge from both professionals and researchers—practice and research. Examples of benefits are prevention of ill health and improved health for the older people. When involving the professionals, their knowledge about practice, their relation to the older people, and insight into their situation may lead to greater understanding of the older peoples’ needs. Professionals may provide a feeling of security and trust to the older people, and more situational information that contributes to learning. The professionals may also enable the older peoples' voice to be heard, ensuring that the research focuses on their needs, which may lead to creation of relevant interventions for the older people. When professionals and researchers collaborate, the research contributes to a systematic approach in practice, and the research forms the basis for improvement work.

The cluster has an average BV of 0.16. The statement with lowest BV (0.04) is statement 54, “Research has effect in practice independently of research results”. Statement 5, “The research is focused on the everyday needs of the older people”, was rated highest in terms of strengthening practice (3.47). Statement 58, “Increased social benefits”, was rated highest in terms of strengthening research (3.43).

## Discussion

The aim of this study was to conceptualize professionals’ involvement in research on ageing and health from the perspective of professionals themselves. Using the GCM method, eight conceptual areas illustrating professionals’ perspectives on involvement in research emerged. A latent qualitative analysis of the conceptual areas resulted in the following three themes: *integrated knowledge benefits older people; prerequisites and professionals’ learning can contribute to development of practice;* and *challenges for professionals*.

### Integrated knowledge benefits older people

The theme *integrated knowledge benefits older people* indicates that the involvement of professionals in research on ageing and health can lead to the integration of knowledge that can benefit older people and their potential for healthy ageing. This is illustrated in the conceptual areas *bridging the gap between practice and research*, *applicable research that benefits practice*, and *research that benefits older people,* which were all rated highest in terms of strengthening research and practice. Integration of knowledge is when different kinds of knowledge are merged and usability is enhanced [42]. In some respects, the professionals hold knowledge that is situational and practical (practical knowledge), and the researchers hold knowledge that is universal and theoretical (scientific knowledge) [9, 42, 43]. Acknowledgement and integration of different ways of knowing and different kinds of knowledge are called for [9, 43] and can contribute to strengthening both practice and research, which can benefit older people. These knowledge forms and the integration of them are not new in healthcare. More than two decades ago, Sackett et al. [44] had already described evidence-based medicine as an integration of different knowledge forms. They emphasized the integration of the best current scientific evidence (scientific knowledge) with the professional’s clinical expertise (practical knowledge) and also the importance of the preferences of the persons receiving the care.

However, there is a risk of simplifying when knowledge is categorized as practical or scientific, since both professionals and researchers use different knowledge forms on a daily basis. McHugh and Walker [43] build on Polanyi’s [45] description of knowledge and describe different forms of knowledge, relating it to practising medicine. They explain how both tacit and explicit knowledge, as well as particular and general knowledge, are used when practising medicine [43]. However, this can apply to all people working in healthcare, since an integration of different knowledge forms is needed to provide the best care for that specific person, in that specific situation. Researchers also use tacit knowledge gained through experience from prior research, and particular knowledge when targeting a specific group of people. The integration of practical knowledge with scientific knowledge is emphasized in several research designs. For example, integration is used as a way to create actionable scientific knowledge in collaborative management research [42], and there is a similar focus on integrating knowledge in other research designs such as integrated knowledge translation (IKT), engaged scholarship, mode 2 research, coproduction, and participatory research, despite the variations in the approaches [46]. The gap between research and practice may be related to the different forms of knowledge and the separation between them [47], where one or the other might be preferred by researchers or professionals. To integrate knowledge is difficult, but important to consider when aiming at development of practice, and it may also benefit older people by enhancing their potential for healthy ageing. However, as this study shows, the prerequisites for collaboration and the possibility to learn from each other are important parts of the process of collaboration.

### Prerequisites and professionals’ learning can contribute to development of practice

The theme *Prerequisites and professionals’ learning can contribute to development of practice* indicates that when the prerequisites facilitate the professional’s involvement in research and their ability to learn, this may lead to a development of practice. This is illustrated in the conceptual areas *prerequisites affecting professionals’ involvement in research*, *professionals’ involvement increases their interest and engagement in R&D*, and *professionals contribute with their knowledge*. When aiming at strengthening healthcare systems by developing new knowledge, the professionals must be involved, since as Nonaka [48] states, “an organisation cannot create knowledge without individuals” (p. 17). Furthermore, Nonaka [48] elaborates on the exchange of tacit and explicit knowledge which can inform each other, and thus create new knowledge. The importance of an iterative dialogue and exchange of tacit and explicit knowledge in the collaboration process when co-creating knowledge is also emphasized. Researchers and professionals may learn from each other when collaborating [4, 49]. A longitudinal, multiple-case study showed that by applying a system theoretical view to an action research project, development and learning occurred at several organizational levels, and this was believed to lead to a sustainable change [50]. However, collaboration is complex, and a dialogue between professionals and researchers is needed to understand each other’s worldview and to integrate the perspectives of research and practice. Hence, acquiring and creating new knowledge is facilitated by the exchange of knowledge in a dynamic process [48]. Looking at the prerequisites for not only involving professionals in research but also facilitating the possibility to integrate knowledge, our study highlights professionals’ need for support from management and their belief in their own expertise and knowledge. A review by King et al. [51] showed that professionals’ lack of confidence in their ability to conduct research was a barrier to their involvement in research projects. Their lack of confidence was found to be related to a lack of skills, competencies, and knowledge of conducting research. However, our study indicates that when professionals are involved in research, they develop a critical approach and acquire an understanding of research.

### Challenges for professionals

The theme *challenges for professionals* indicates that the involvement of professionals in research causes them to encounter several challenges. This is illustrated in the conceptual areas *challenges for professionals in relation to practice* and *challenges for professionals in relation to research.* Both conceptual areas were rated low in terms of strengthening research and practice, making them even more important to be aware of when involving professionals. Challenges in the professionals’ relation to practice and research need attention and must be dealt with by both researchers and professionals in order to facilitate the involvement of the professionals. Other studies have found that professionals can perceive it as challenging to be involved because of a lack of time [52] or a lack of funding for the time they invest [53]. Our study shows that professionals experience that when involved in research, they might be assigned extra tasks, which takes time from other tasks in their daily work. Other challenges the professionals may face, illustrated in our study, are of a cultural nature. Professionals can experience standing between two groups of people—their colleagues and researchers—having different worldviews and values. Still, professionals involved can play an essential role in research projects, acting as interpreters and coordinators between researchers and other professionals. However, Bowen et al. [54] finds that not only the differences between the culture of research and the culture of practice influence the collaboration, but also different cultures in organizational cultures, making collaboration between professionals difficult.

Furthermore, within the conceptual area *challenges for professionals in relation to research*, it is stated that professionals need knowledge about scientific concepts. However, to what extent professionals have to learn about research and be embedded in the worldview of research are matters for debate. A core aspect of collaboration is that people with different perspectives come together to merge perspectives, and use different kinds of knowledge to create a more comprehensive understanding of the issue at target [55]. This study shows that collaboration and bringing together perspectives from research and practice can have implications for practice by making research more situational, leading to applicable research that benefits practice. Also, when the research projects become more anchored in practice, it may lead to faster implementation of research results and facilitation of the dissemination of the research.

However, health systems are complex and consist of several different professionals interacting with each other and the older people and their relatives. Hence, it is important to also investigate the involvement of professionals from different levels, such as micro-, meso-, exo-, and macro-levels [15], and also to investigate the possibility of involving older people [56] as well as carers [57] in research. Furthermore, “systems thinking” illustrates the complexity of diffusion, dissemination, and exchange of knowledge [58] and stresses the importance of collaboration between researchers and professionals, of having multidisciplinary teams, and of involving people from outside academia in research [59]. Professionals at different levels, in a system theoretical perspective, all play an important role in facilitating healthy ageing for older people, but more knowledge is needed about who should be involved as well as when the involvement in research should occur, in what form and why, depending on the aim of the project [60]. Summing up, the need for actions to improve older people’s potential for healthy ageing [3] require collaboration between researchers and different stakeholders at multiple levels and in multiple sectors so that the various parties can exchange and integrate knowledge.

### Methodological considerations

Given the complex area investigated, the use of a mixed method that provides both breadth and depth was considered relevant [30]. In the exploratory sequential approach, the qualitative phase precedes and informs the quantitative phase as in GCM studies; however, the method differs to some extent. For example, in the exploratory sequential design, the qualitative phase often results in themes in which quantitative features will be tested, such as when using the data from the qualitative phase to develop items for an instrument which can then be tested quantitatively [30]. In GCM studies, the quantitative data collection contributes to the resulting conceptual areas, and therefore the method not only builds on but also integrates both data collection and data interpretation.

Professionals who were interested in participating in the study but were unable to take part in a brainstorming session only participated in the organizing step. This is acceptable in GCM studies, since the method allows the same people or different people to participate in the brainstorming sessions and the organizing step (the qualitative and the quantitative phase), depending on the sampling strategy and the logistics of the project [34]. GCM is not only a mixed method, but also a method that actively involves the participants in some of the steps of the research process, and it can be seen as rather time-consuming by the participants. More than half of the professionals who were asked to participate declined, mainly due to time constraints. A few participants in the study dropped out after the brainstorming and during the organizing step, which may also have been related to time constraints. The professionals who participated had a wide range of experience of being involved in research, extending from consultation to collaboration. However, this study does not differentiate between the different intensities of involvement, which may be a limitation for the usability of the results.

GCM is considered a reliable and valid research method [35], but when involving people from outside academia, such as professionals, in the research, it is important to ensure clear information to the participants about the aim and how to conduct the different steps. The participants received written and verbal information about the study and how to conduct the different steps they were involved in, and that they could always contact the authors if they had questions about the study or the involvement. Some of the participants expressed that they found it difficult to sort the statements, and some contacted the first author with questions or to request clarification of how to conduct the sorting. These queries concerned technical issues, since the sorting was done through a webpage, and practical issues around how to sort the statements.

The focus of this study was professionals’ involvement in research projects on ageing and health, but the results are believed to be transferable to other areas, since several aspects of collaboration between researchers and professionals are probably the same whatever the area of research. By solely including the professional’s perspective, the study in itself is limited, but it adds to the knowledge about what the involvement of professionals can lead to, investigated from the researcher’s perspective [4].

To enhance the trustworthiness of the study, special consideration was given to the qualitative analysis. For example, all the authors participated in the synthesizing of statements as well as in the latent qualitative analysis to enhance dependability. Another example is the 432 statements gathered through brainstorming sessions, which were reviewed and synthesized into a final list of 80 statements indicating saturation of the investigated area. Regarding synthesizing of the statements, in order to enhance credibility, the participants’ own formulations were emphasized, the audit trail of the process was documented, and member checks were conducted.

Regarding the implications of the results, they may be of interest in the development of practical guidance of areas to consider when professionals and researcher collaborate. For example, when researchers and professionals collaborate in research projects, they need to reflect upon and discuss challenges the professionals could meet and how to handle these challenges to facilitate a successful involvement. Also, prerequisites and professionals’ possibilities for learning when involved in research need more attention when aiming at developing practice to benefit older people. By adding to the knowledge of the area, the results may be of interest when developing instruments to measure involvement and when developing education for both professionals and researchers about collaboration.

## Conclusion

This study explored and conceptualized professionals’ own perspectives concerning their experiences of being involved in research, contributing an illustration of conceptual areas of what the involvement of professionals in research can lead to. The study showed that professionals’ involvement in research on ageing and health may lead to knowledge being integrated and to professionals learning through the involvement and contributing to the development of practice. However, the study also showed that there are challenges that need to be handled when professionals are involved in research. The study illustrates the need for different knowledge forms and the importance of integrating knowledge when aiming at bridging the gap between research and practice. This study can be useful for improving the understanding of involvement and for optimizing the involvement of professionals in research. However, the challenges professionals meet when involved need more investigation to gain knowledge about how to work with them as well as how to improve the prerequisites for the professional’s involvement. In order to achieve this, research is needed to explore the managers’ perspective on the involvement of their staff in research studies.

## Data Availability

The data sets used and/or analysed during the current study are available from the corresponding author on reasonable request.

## Abbreviations

BV: Bridging value
GCM: Group concept mapping
MDS: Multidimensional scaling
R&D: Research and development
